# Reply to Michał Bogdziewicz et al.: Research on the summer solstice needs falsifiable predictions

**DOI:** 10.1073/pnas.2516303122

**Published:** 2025-08-18

**Authors:** Victor Van der Meersch, E. M. Wolkovich

**Affiliations:** ^a^Department of Forest and Conservation Sciences, Faculty of Forestry, University of British Columbia, Vancouver, BC V6T 1Z4, Canada

We showed that summer solstice appears as an optimal time to transition between major physiological growth and reproductive phases (1). Given a trade-off between predicting total and remaining thermal heat, summer solstice was optimal on average for Europe and North America, but with local variation. Bogdziewicz et al. interpret our results as invalidating the potential role of solstice in mast seeding (2), but we suggest they have misinterpreted our work. We explicitly considered whether solstice may control the timing of mast seeding, and our results do not invalidate this hypothesis. We do, however, consider the potential role of other drivers and raise questions about how to differentiate between them given strong covariation (1).

Bogdziewicz et al. argue that summer solstice is a better cue for masting than temperature-related cues because it allows for greater synchrony across individuals (2), whereas we highlight spatial variation in a trade-off based on temperature accumulation. We do not disagree that we show important variability across space. We question, however, their conclusions because: i) in many masting trees, the strength of synchrony among individuals decays with distance, inconsistent with cuing directly to summer solstice (3), and ii) the strong covariation between summer solstice and balance in annual heat accumulation makes it difficult to reject either as the primary cue—a major message of our paper. Showing a masting pattern consistent with cuing to solstice is not the same as demonstrating trees cue to solstice.

Our work highlights the challenge in differentiating between cues. Current studies on whether the summer solstice acts as “celestial starting gun” that opens a “temperature sensing window” has focused on a statistical correlation between an annual metric of seed production and daily temperature to show a shift in the correlation may occur near the summer solstice on average, but this timing similarly varies across space [(4), figure 1 in ref. 2)]. These results echo ours (1) and thus we are confused as to where Bogdziewicz et al. see a discrepancy between these findings and ours. Identifying what level of variation invalidates the solstice-as-cue hypothesis seems an important question, especially as results from summer solstice research for both masting (2, 4) and growth (5) show the statistically most likely date before or after the solstice by one or more weeks.

Developing falsifiable predictions to test the role of the solstice—and other cues—in driving transitions between major physiological phases is important for fundamental science and forecasting. Identifying the spatial scale of synchrony that maximizes fitness based on different economy of scale hypotheses for masting (6) could rule out some cues. We hope more efforts that build from ecoevolutionary and climatological perspectives (1) can provide clearer predictions and stronger evidence, especially as bounded systems [e.g., the growing season, (7, 8)] and natural climatological trends often lead to interesting patterns with multiple competing explanations that make very different predictions (9, 10).
